# Malnutrition Contributes to Low Lymphocyte Count in Early-Stage Coronavirus Disease-2019

**DOI:** 10.3389/fnut.2021.739216

**Published:** 2022-01-06

**Authors:** Kai Zhang, Weidong Qin, Yue Zheng, Jiaojiao Pang, Ning Zhong, Jianchun Fei, Yu Li, Xiangdong Jian, Xinguo Hou, Zhao Hu, Chen Li, Hao Wang, Yuguo Chen

**Affiliations:** ^1^The Key Laboratory of Cardiovascular Remodeling and Function Research, Chinese Ministry of Education, Chinese National Health Commission and Chinese Academy of Medical Sciences, The State and Shandong Province Joint Key Laboratory of Translational Cardiovascular Medicine, Department of Cardiology, Qilu Hospital, Cheeloo College of Medicine, Shandong University, Jinan, China; ^2^Department of Critical Care Medicine, Qilu Hospital of Shandong University, Jinan, China; ^3^Shandong Provincial Clinical Research Center for Emergency and Critical Care Medicine, Institute of Emergency and Critical Care Medicine of Shandong University, Chest Pain Center, Qilu Hospital of Shandong University, Jinan, China; ^4^Department of Emergency Medicine, Qilu Hospital of Shandong University, Jinan, China; ^5^Key Laboratory of Emergency and Critical Care Medicine of Shandong Province, Key Laboratory of Cardiopulmonary-Cerebral Resuscitation Research of Shandong Province, Shandong Provincial Engineering Laboratory for Emergency and Critical Care Medicine, Qilu Hospital of Shandong University, Jinan, China; ^6^Department of Gastroenterology, Qilu Hospital of Shandong University, Jinan, China; ^7^Department of Anesthesiology, Qilu Hospital of Shandong University, Jinan, China; ^8^Department of Respiratory and Critical Care Medicine, Qilu Hospital of Shandong University, Jinan, China; ^9^Department of Poisoning and Occupational Diseases, Qilu Hospital of Shandong University, Jinan, China; ^10^Department of Endocrinology, Qilu Hospital of Shandong University, Jinan, China; ^11^Department of Nephrology, Qilu Hospital of Shandong University, Jinan, China; ^12^Department of Pharmacology, School of Basic Medical Science, Shandong University, Jinan, China

**Keywords:** malnutrition, COVID-19, lymphocyte, SARS-CoV-2, lymphopenia

## Abstract

**Background and Aim:** Lymphocytes play an important role in fighting severe acute respiratory syndrome coronavirus 2 (SARS-CoV-2) infections. Low total lymphocyte count (TLC), which contributes to poor clinical outcomes, is common in persons with coronavirus disease 2019 (COVID-19). The current explanation for the cause of low TLC is that it is directly related to the invasiveness of SARS-CoV-2, which attacks lymphocytes. We hypothesized that malnutrition contributes to the development of low TLC in early-stage COVID-19.

**Methods:** We prospectively enrolled 101 patients with confirmed COVID-19. On their first day of hospitalization, we collected baseline and laboratory data, including clinical symptoms; the Sequential Organ Failure Assessment, Nutrition Risk Screening 2002 and Subjective Global Assessment were used to assess the malnutrition status of the patients. Multivariable logistic regression was used to identify independent risk factors for low TLC and severe COVID-19.

**Results:** Malnutrition was associated with lower TLC in COVID-19. Fifty-nine (58.4%) of the patients showed low TLC, 41 (40.6%) were at risk for malnutrition, and 18 of them were malnourished. Low TLC was an independent risk factor for severe COVID-19. Compared to patients with normal TLC, those with low TLC more often presented with anorexia, malnutrition, higher SOFA scores (*P* < 0.05) and comorbidities (diabetes and malignancies). Malnutrition (OR: 3.05, 95% CI: 1.5–6.19, *P* = 0.006) and SOFA scores (OR: 1.51, 95% CI: 1.04-2.43, *P* = 0.042) were identified as independent risk factors for low TLC.

**Conclusions:** Malnutrition was common among our patients with early-stage COVID-19, and it contributed to the occurrence of low TLC.

## Introduction

Coronavirus disease 2019 (COVID-19), which is caused by severe acute respiratory syndrome coronavirus 2 (SARS-CoV-2), continues its rapid spread globally. A lymphocyte is a type of immune cell that plays an important role in fighting SARS-CoV-2 infection, and low total lymphocyte count (TLC) is common in COVID-19, ranging from 35 to 82% of patients (1, 2). Low TLC also contributes to poor clinical outcomes (2). Non-survivors showed severe lymphopenia over time (3). Increasing the TLC level may be a key therapeutic target for these infections, but until now, the cause of low TLC has been thought to be directly related to the invasive nature of SARS-CoV-2, which attacks immune cells (1, 4).

However, the cause of lymphopenia could be multifactorial, and malnutrition is a main contributor (5). Intensive care unit (ICU) stay, polymorbidity, and older age are associated with high risk for malnutrition, representing a relevant risk factor for higher morbidity in COVID-19 (6). The high incidence of gastrointestinal symptoms in COVID-19 may further increase malnutrition risk (7, 8). A recent study reported that 52.7% of elderly patients with COVID-19 are malnourished, and that 27.5% is at risk for malnutrition (9). Lymphopenia may occur when there is lack of protein and other nutrients that are necessary to produce enough lymphocytes. Significant decrease in TLC has been observed in elderly patients who are malnourished (9), indicating a possible correlation between TLC and malnutrition in persons with COVID-19. In this study, we aimed to establish a multivariable model and determine whether malnutrition is an independent risk factor for low TLC in COVID-19. Another goal of this study was to produce evidence for the pathogenesis of lymphopenia in early-stage COVID-19 and demonstrate the feasibility of elevating TLC levels through nutrition management as a treatment for affected patients.

## Materials and Methods

We prospectively enrolled adult patients admitted to the COVID-19 infection ward of a designated hospital in Wuhan who have confirmed COVID-19 from February 6 to March 20, 2020. On the first day of hospitalization, baseline data, clinical symptoms from disease onset to hospitalization, and laboratory data of the patients were collected. The Sequential Organ Failure Assessment (SOFA)^1^, which measures the degree of dysfunction of six organ systems (respiratory, liver, cardiovascular, renal, coagulation, and central nervous system), was conducted. The Nutrition Risk Screening 2002 (NRS-2002) was performed to identify patients at risk for malnutrition. Patients with an NRS-2002 score ≥ 3 were further assessed using the Subjective Global Assessment (SGA) (6). Malnutrition status was divided into three categories: (1) no risk for malnutrition (NRS-2002 score <3), (2) risk for malnutrition (NRS-2002 score ≥ 3 with Subjective Global Assessment [SGA] A classification) and (3) malnourished (NRS-2002 score ≥ 3 and SGA B or C classification). Hospitalized patients were excluded from the study if they (1) were lost to follow-up or had insufficient information; or (2) history of cardiac arrest, death within 24 h or length of stay of less than 72 h, or with do-not-resuscitate orders. To verify the correlation between malnutrition and low TLC in non-hospitalized patients, the non-hospitalized patients with sufficient clinical information (such as NRS-2002 scores and results of routine blood tests) were enrolled at the same time. Their malnutrition status was classified as no risk for malnutrition (NRS-2002 score <3) and risk for malnutrition (NRS-2002 score ≥ 3).

Data on the use of lymphopenia-inducing drugs (corticosteroids, cytotoxic drugs, thymic hormones, and interferons) (10) within 2 weeks and coexisting comorbidities related to lymphopenia (5) were also collected as confounding factors. The study was approved by the ethics committee of Qilu Hospital. Clinical outcomes of the hospitalized patients consisted of acute respiratory distress syndrome (ARDS), severe COVID-19, ICU care, and mortality. The outcomes of ARDS and severe COVID-19 were defined according to criteria from the World Health Organization (WHO) (11, 12). Mortality was defined as death, and ICU care was defined as the day of admittance to the ICU during the in-hospital care of a patient.

SPSS 16.0 (SPSS Inc, Chicago, IL, United States) was used for data analysis. Multivariable logistic regression analysis was performed to identify independent risk factors associated with low TLC and severe COVID-19, and odds ratios (ORs) and 95% confidence intervals (CIs) were estimated. Variables with *P* ≤ 0.2 on the univariate analyses were included in the multivariable model. *P* < 0.05 was considered statistically significant. Univariate logistic regression was used to assess the consistency of the relationships between the categories of malnutrition status and low TLC with clinical outcomes.

## Results

A total of 101 cases were enrolled. As shown in Table 1, the mean age of the enrolled participants is 65.3 ± 13 years, and 59.4% are male. The mean SOFA score on the first day of hospitalization was 2.4 ± 1.3, and 59 (58.4%) of the patients showed low TLC on admission. The most common comorbidities were hypertension (*n* = 26, 25.7%), cardiovascular disease (*n* = 13, 12.9%), and diabetes (*n* = 13, 12.9%). A total of 41 (40.6%) patients were at risk for malnutrition (NRS-2002 score ≥ 3); 18 of them were malnourished, and 23 were at risk for malnutrition but were not malnourished. As shown in Supplementary Figure 1, low TLC and malnourishment are associated with most worst clinical outcomes. Low TLC, instead of malnourishment, was identified as an independent risk factor for severe COVID-19 (Supplementary Tables 1, 2) using the multivariate logistic model.

**Table 1:** Univariate analysis of the risk factors for low TLC in hospitalized patients with early-stage COVID-19.

**Characteristics**	**Total (*N* = 101)**	**Low TLC** **(*N* = 59)**	**Normal TLC (*N* = 42)**	***P* value**
Age, years	65.3 ± 13.0	66.6 ± 14.3	63.6 ± 10.8	0.256
Sex				
Male	60 (59.4)	35 (59.3)	25 (59.5)	
Female	41 (40.6)	24 (40.7)	17 (40.5)	0.984
Lymphocyte count, ×10^9^/L	1.12 ± 0.59	0.75 ± 0.22	1.64 ± 0.54	-
**Comorbidities**				
Hypertension	26 (25.7)	16 (27.1)	10 (23.8)	0.708
Cardiovascular disease	13 (12.9)	7 (11.9)	6 (14.3)	0.955
Diabetes	13 (12.9)	10 (16.9)	3 (7.1)	0.147
Cerebrovascular disease	4 (4.0)	3 (5.1)	1 (2.4)	0.866
Chronic obstructive pulmonary disease	3 (3.0)	2 (3.4)	1 (2.4)	0.764
Chronic liver disease	2 (2.0)	2 (3.4)	0 (0.0)	0.631
Chronic kidney disease	4 (4.0)	4 (6.8)	0 (0.0)	0.228
Comorbidities related to lymphopenia				
Malignancy	11 (10.9)	9 (15.3)	2 (4.8)	0.095
Lupus	1 (1.0)	1 (1.7)	0 (0.0)	0.864
Rheumatoid arthritis	2 (2.0)	1 (1.7)	1 (2.4)	0.631
Others^a^	0 (0.0)	0 (0.0)	0 (0.0)	-
**Clinical symptoms**				
Fever	93 (92.1)	55 (93.2)	38 (90.5)	0.615
Cough	66 (65.3)	40 (67.8)	26 (61.9)	0.540
Dyspnea	33 (32.7)	21 (35.6)	11 (26.2)	0.317
Anorexia	38 (37.6)	27 (45.8)	11 (26.2)	**0.045**
Diarrhea	14 (13.8)	10 (16.9)	4 (9.5)	0.287
Vomiting	9 (9.0)	6 (10.2)	3 (7.1)	0.864
**Use of drug that may induce lymphopenia within 2 weeks before hospitalization**				
Corticosteroid	9 (8.9)	6 (10.2)	3 (7.1)	0.864
Thymic hormones	5 (5.0)	3 (5.1)	2 (4.8)	0.941
Cytotoxic drugs	3 (3.0)	2 (3.4)	1 (2.4)	0.764
Interferon	2 (2.0)	1 (1.7)	1 (2.4)	0.631
Others^b^	0 (0.0)	0 (0.0)	0 (0.0)	-
**Malnutrition status**				
1 No risk for malnutrition	60 (59.4)	26 (44.1)	34 (81.0)	**<0.001**
2 Risk for malnutrition	23 (22.7)	17 (28.8)	6 (14.2)	0.086
3Malnourished	18 (17.8)	16 (27.1)	2 (4.8)	**0.004**
SOFA score	2.4 ± 1.3	2.7 ± 1.5	2.0 ± 0.8	**0.012**
**Outcomes**				
Severe COVID-19	54 (53.4)	37(62.7)	17 (42.5)	**0.027**
ARDS	38 (37.6)	27 (45.8)	11 (26.2)	**0.045**
ICU care	17 (16.8)	13 (22.0)	4 (9.5)	**0.098**
Death	5 (4.9)	4 (6.8)	1 (2.4)	0.315

*Data are reported as n (%) or mean ± standard deviation (SD). TLC, total lymphocyte count; COVID-19, coronavirus disease 2019; SOFA, Sequential Organ Failure Assessment; ARDS, acute respiratory distress syndrome; ICU, intensive care unit. The cut-off point for low TLC and normal TLC is 1.1 × 10^9^/l. a. including aplastic anemia, human immunodeficiency virus (HIV), hypersplenism, myelodysplastic syndrome, and tuberculosis. b. including monoclonal antibodies, cimetidine, and opioids.*

*Bold values are p < 0.05, which are considered statistically significant*.

Compared to patients with normal TLC, those with low TLC more often presented with anorexia from the onset of the disease. They were more often malnourished and had higher SOFA score but less often well-nourished (*P* < 0.05, Table 1). The univariate analysis of the risk factors for TLC identified five indicators (i.e., comorbid diabetes and malignancy, anorexia, higher SOFA score, and malnutrition), which were included in the multivariate analysis. A similar trend in malnutrition was found among the cases that were not hospitalized (Supplementary Table 3).

The multivariate logistic regression identified the SOFA score (OR: 1.51, 95% CI: 1.04–2.43, *P* = 0.042) and a trend toward malnutrition (OR: 3.05, 95% CI: 1.5–6.19, *P* = 0.006) as independent risk factors for low TLC (Table 2). Compared to patients who were not at risk for malnutrition (set as a reference), the risk for low TLC increased 3.67- and 7.63-fold in patients who were at risk for malnutrition and those who were malnourished.

**Table 2:** Multivariate logistic regression analysis of the risk factors for low TLC in hospitalized patients with early-stage COVID-19.

**Characteristics**	**Univariate analysis**		**Multivariable analysis**	
	**OR (95%CI)**	***P* value**	**OR (95%CI)**	***P* value**
Diabetes	2.65 (0.68–10.31)	1.159		
Malignancy	3.60 (0.74–17.61)	0.114		
Anorexia symptom	2.38 (1.01–5.61)	0.048		
SOFA score	1.65 (1.09–2.50)	0.018	1.51 (1.04–2.43)	0.042
Malnutrition status				
1 No risk for malnutrition	Reference	-	Reference	-
2 Risk for malnutrition	3.71 (1.28–10.71)	0.016	3.67 (1.24–10.82)	0.019
3 Malnourished	10.46 (2.21–49.59)	0.003	7.63 (1.55–35.58)	0.012
*P* value for trend	3.39 (1.71–6.71)	0.002	3.05 (1.50–6.19)	0.006

*TLC, total lymphocyte count; OR, odds ratio; CI, confidence interval; SOFA, Sequential Organ Failure Assessment*.

## Discussion

Our study found that malnutrition was common among our patients with early-stage COVID-19, and that it contributed to their low TLC. The data suggest that malnutrition is another important cause of lymphopenia, in addition to the invasive nature of SARS-CoV-2, which attacks immune cells.

Previous studies have identified lymphopenia as a significant prognostic marker for COVID-19 and TLC as a variable associated with disease severity and mortality (4, 13, 14). Appropriate functions of lymphocytes, such as cytotoxicity, antibody production, and immune response regulation, play critical roles in restricting SARS-CoV-2 infection. Lymphopenia is a hallmark of predisposition to severe disease, with incidence of about 85% in patients with severe COVID-19 (15). In this study, low TLC was also verified to be an independent risk factor for severe COVID-19 in hospitalized patients. An understanding of the pathogenesis of low TLC may facilitate the development and testing of therapeutic targets for COVID-19 management.

Malnutrition has been observed frequently in persons with infectious diseases such as COVID-19 (9, 16, 17). Thus, nutritional support is probably a reasonable approach to improve their prognosis (18, 19). The causes of malnutrition in COVID-19 are multifactorial. Polymorbidity, older age (6), gastrointestinal symptoms (7, 8), and catabolism stress induced by infection, which are commonly found in COVID-19, may contribute to patients' deteriorated nutritional status in COVID-19. Sufficient protein and nutrients are necessary to produce lymphocytes, as protein–energy malnutrition leads to reduced production of lymphocytes (20). TLC is also a popular serum marker, which may be useful for determining nutritional status. Levels of TLC have been found to vary with the degree of malnutrition; for example, TLV levels <1,500/mm^3^ show a strong correlation with malnutrition, and levels <900/mm^3^ reflected severe malnutrition (21, 22). Consequently, immune function is affected, thereby increasing the risk of infection due to impaired cell-mediated immunity and cytokine, complement, and phagocyte function (23). Ensuring adequate nutrition in early-stage COVID-19 might increase the level of TLC and improve immune function and prognosis, but it needs to be verified by further intervention studies. Previous studies have found that malnutrition is associated with worse clinical outcomes (9, 16, 17), and nutritional support probably improves the prognosis (18, 19) of infectious diseases (including COVID-19). In this study, we also found that malnourishment was associated with the worse clinical outcomes in the univariate model (Supplementary Figure 1). However, malnutrition was not found to be an independent risk factor for severe COVID-19 in the multivariate analysis. Considering the significant correlation between malnutrition and low TLC, the prognostic value of malnutrition, with respect to COVID-19, may be partly attributable to its contribution to lymphopenia.

In this study, SOFA score was identified as an independent risk factor for low TLC, and the reason was probably related to the invasiveness of SARS-CoV-2. Infection with SARS-CoV-2 can damage multiple organ systems, including the respiratory (24), liver (25), cardiovascular (26), renal (27), coagulation (28), and central nervous systems (29). Given that SOFA score is calculated based on the degree of dysfunction of the above organ systems, higher SOFA score may indicate higher level of invasiveness of SARS-CoV-2 and damage to organ systems. Furthermore, SOFA score has been found to be associated with poor prognosis for COVID-19 (30).

Our study had some limitations. First, it was limited by the observational nature of the data. Further intervention research is needed regarding the effectiveness of nutrition support on lymphocyte count in patients with COVID-19. Second, because of the small sample size of this study, a multivariate model was not established to determine the association of lymphopenia and malnutrition with all clinical outcomes. Further study with a large sample size is warranted.

Our study indicated that malnutrition was common among our patients with early-stage COVID-19, and that it is an important but generally unrecognized cause of lymphopenia. Ensuring adequate nutrition in early-stage COVID-19 may prevent low TLC; therefore, this hypothesis needs to be verified through further intervention studies.

## Data Availability Statement

The raw data supporting the conclusions of this article will be made available by the authors, without undue reservation.

## Ethics Statement

The studies involving human participants were reviewed and approved by the Ethics Committee of Qilu Hospital. The patients/participants provided their written informed consent to participate in this study.

## Author Contributions

HW, YC, and YL concept and design. All authors: acquisition, analysis, or interpretation of the data. HW and NZ drafting of the manuscript. HW and JF statistical analysis. HW and YC obtained funding. XH and ZH contributed to the collection of additional data and analysis in the revision. All authors approved the final version of the manuscript and are responsible for the content.

## Funding

This study was supported by grants from the National Natural Science Foundation of China (81873927, 82072231), Key Research Plan of Shandong Province (2020SFXGFY03), Taishan Scholars Program of Shandong Province (tsqn202103165), Clinical Research Center of Shandong University (2020SDUCRCC013), and China Postdoctoral Science Foundation (2018M632685).

## Conflict of Interest

The authors declare that the research was conducted in the absence of any commercial or financial relationships that could be construed as a potential conflict of interest.

## Publisher's Note

All claims expressed in this article are solely those of the authors and do not necessarily represent those of their affiliated organizations, or those of the publisher, the editors and the reviewers. Any product that may be evaluated in this article, or claim that may be made by its manufacturer, is not guaranteed or endorsed by the publisher.

## Footnotes

^1^ https://www.mdcalc.com/sequential-organ-failure-assessment-sofa-score#pearls-pitfalls
