# Supplementary material for: Malnutrition Contributes to Low Lymphocyte Count in Early-Stage Coronavirus Disease-2019
Source: Front Nutr. 2022 Jan 6;8:739216. doi: 10.3389/fnut.2021.739216 (PMC8770860; doi:10.3389/fnut.2021.739216)
Supplement: Supplementary file 1 [file Data_Sheet_1.DOCX]

**Supplementary Materials**

**Malnutrition Contributes to Low Lymphocyte Count in Early-stage Coronavirus Disease 2019**

Kai Zhang, Weidong Qin, Yue Zheng, Jiaojiao Pang, Ning Zhong, Jianchun Fei, Yu Li, Xiangdong Jian, Zhao Hu, Chen Li, Hao Wang, and Yuguo Chen


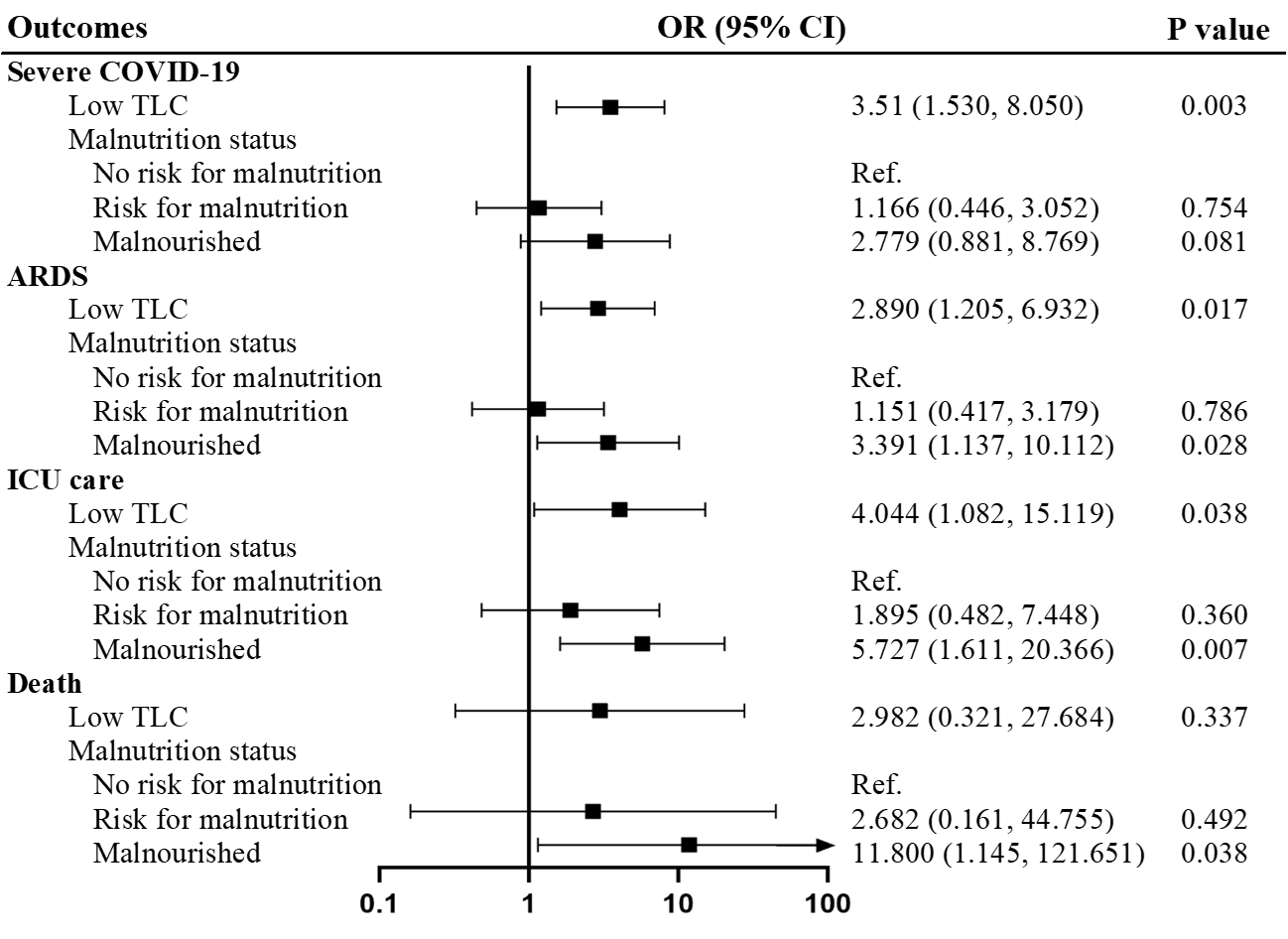


**Figure S1** The relationship between the categories of malnutrition status and low TLC with clinical outcomes in in hospitalized patients of early-stage COVID-19

COVID-19, coronavirus disease 2019; TLC, total lymphocytes count; ARDS, acute respiratory distress syndrome; ICU, intensive care unit; OR, odd ratio; CI, confidential interval.

**Table S1.** Multivariate logistic regression analysis of the risk factors for severe coronavirus disease 2019 in hospitalized patients (including low TLC)

| **Characteristics** | **Univariate analysis** | | **Multivariable analysis** | |
| --- | --- | --- | --- | --- |
|  | **OR (95%CI)** | ***P* value** | **OR (95%CI)** | ***P* value** |
| Low TLC | 3.51 (1.53-8.05) | 0.003 | 3.04 (1.07-8.66) | **0.037** |
| Age | 1.04 (1.01-1.07) | 0.037 | 1.00 (0.96-1.04) | 0.907 |
| Cardiovascular disease | 3.75 (0.98-14.39) | 0.054 | 3.16 (0.35-28.30) | 0.303 |
| Diabetes | 2.25 (0.64-7.86) | 0.099 | 1.32 (0.23-7.58) | 0.759 |
| Diarrhea symptom | 5.76 (1.21-27.49) | 0.028 | 3.90 (0.36-42.34) | 0.759 |
| SOFA score | 5.35 (2.66-10.76) | 0.001 | 5.07 (2.31-11.14) | **0.001** |

TLC, total lymphocyte count; SOFA, Sequential organ failure assessment.

**Table S2.** Multivariate logistic regression analysis of the risk factors for severe coronavirus disease 2019 in hospitalized patients (including malnutrition status)

| **Characteristics** | **Univariate analysis** | | **Multivariable analysis** | |
| --- | --- | --- | --- | --- |
|  | **OR (95%CI)** | ***P* value** | **OR (95%CI)** | ***P* value** |
| Malnutrition status |  |  |  |  |
| 1 No risk for malnutrition | Reference | - | Reference | - |
| 2 Risk for malnutrition | 1.17 (0.45-3.05) | 0.754 | 1.56 (0.30-8.16) | 0.596 |
| 3 Malnourished | 2.78 (0.88-8.77) | 0.081 | 1.30 (0.21-8.07) | 0.776 |
| Age | 1.04 (1.01-1.07) | 0.037 | 1.01 (0.96-1.05) | 0.791 |
| Cardiovascular disease | 3.75 (0.98-14.39) | 0.054 | 1.61 (0.20-13.07) | 0.656 |
| Diabetes | 2.25 (0.64-7.86) | 0.099 | 1.40 (0.26-7.51) | 0.698 |
| Diarrhea symptom | 5.76 (1.21-27.49) | 0.028 | 2.24 (0.24-21.03) | 0.480 |
| SOFA score | 5.35 (2.66-10.76) | 0.001 | 5.33 (2.42-11.72) | **0.001** |

SOFA, Sequential organ failure assessment.

**Table S3.** Univariate analysis of the risk factors for low total lymphocyte count (TLC) in non-hospitalized patients of coronavirus disease 2019

| **Characteristics** | **Total**  **(N = 39)** | **Low TLC**  **(N = 8)** | **Normal TLC**  **(N = 31)** | ***P* value** |
| --- | --- | --- | --- | --- |
| Age, years | 54.5 ± 7.7 | 57.2 ± 9.0 | 53.8 ± 7.2 | 0.235 |
| Sex |  |  |  |  |
| Male | 18 (46.2) | 4 (50.0) | 14 (45.2) |  |
| Female | 21 (53.8) | 4 (50.0) | 17 (54.8) | 0.807 |
| **Comorbidities** |  |  |  |  |
| Hypertension | 5 (15.4) | 2 (25.0) | 3 (9.7) | 0.574 |
| Cardiovascular disease | 2 (5.1) | 1 (12.5) | 1 (3.2) | 0.872 |
| Diabetes | 3 (7.7) | 1 (12.5) | 2 (6.5) | 0.864 |
| Cerebrovascular disease | 2 (5.1) | 0 (0.0) | 2 (6.4) | 0.872 |
| Chronic obstructive pulmonary disease | 1 (2.6) | 1 (12.5) | 0 (0.0) | 0.459 |
| Chronic liver disease | 1 (2.6) | 0 (1.7) | 1 (3.2) | 0.607 |
| Chronic kidney disease | 1 (2.6) | 1 (12.5) | 0 (0.0) | 0.459 |
| Comorbidities related to lymphopenia |  |  |  |  |
| Malignancy | 2 (5.1) | 1 (12.5) | 1 (3.2) | 0.872 |
| Rheumatoid arthritis | 1 (2.6) | 0 (0.0) | 1 (3.2) | 0.607 |
| Others ^a^ | 0 (0.0) | 0 (0.0) | 0 (0.0) | - |
| **Clinical symptoms** |  |  |  |  |
| Fever | 33 (84.6) | 6 (75.0) | 27 (87.1) | 0.398 |
| Cough | 25 (64.1) | 5 (62.5) | 20 (64.5) | 0.916 |
| Dyspnea | 5 (12.8) | 2 (25.0) | 3 (9.7) | 0.574 |
| Anorexia | 7 (17.9) | 3 (37.5) | 4 (12.9) | 0.272 |
| Diarrhea | 3 (7.7) | 1 (12.5) | 2 (6.5) | 0.864 |
| Vomiting | 1 (2.6) | 1 (12.5) | 0 (0.0) | 0.459 |
| **Use of drug that may induce lymphopenia within 2 weeks before hospitalization** |  |  |  |  |
| Corticosteroid | 2 (5.1) | 1 (12.5) | 1 (3.2) | 0.872 |
| Thymic hormones | 1 (2.6) | 0 (1.7) | 1 (3.2) | 0.607 |
| Cytotoxic drugs | 0 (0.0) | 0 (0.0) | 0 (0.0) | - |
| Interferon | 1 (2.6) | 0 (0.0) | 1 (3.2) | 0.607 |
| Others ^b^ | 0 (0.0) | 0 (0.0) | 0 (0.0) | - |
| **Malnutrition status** |  |  |  |  |
| 1 No risk for malnutrition | 32 (82.1) | 4 (50.0) | 28 (90.3) |  |
| 2 Risk for malnutrition | 7 (17.9) | 4 (50.0) | 3 (9.7) | **0.033** |

Data are n (%) or mean ± SD.

a, included lupus, aplastic anemia, HIV, hypersplenism, myelodysplastic syndrome, tuberculosis.

b, including monoclonal antibodies, cimetidine and opioids.
